# Balancing Anesthesia in a Child With Mitochondrial Disease: A Case Report

**DOI:** 10.7759/cureus.70756

**Published:** 2024-10-03

**Authors:** Akilandeswari Manickam, Kadhij Fathima, Vatsala A Bagri, Abi Vignesh Prabu Ganesh Prabu, Vishnu Priya R.

**Affiliations:** 1 Department of Anaesthesiology, Sri Ramachandra Institute of Higher Education and Research, Chennai, IND

**Keywords:** case report, general anaesthesia, mitochondrial disorder, oxidative phosphorylation deficiency type 3, paediatric

## Abstract

Anesthetic management of patients with mitochondrial disease requires an in-depth knowledge and understanding of its pathophysiology to ensure the safe conduct of anesthesia. Our case report illustrates the importance of careful anesthetic planning and execution for a four-year-old undergoing full mouth rehabilitation. It is essential to avoid factors that increase metabolic stress, such as prolonged fasting, hypoglycemia, postoperative nausea, hypothermia, acidosis, hypovolemia, and ischemic or hypoxic events. Balanced general anesthesia was achieved by using incremental doses of anesthetics, narcotics, and muscle relaxants, all selected to minimize any potential impact on mitochondrial function. The perioperative period was uneventful.

## Introduction

Children with mitochondrial disease exhibit unique anesthetic challenges during the perioperative period, necessitating careful monitoring with judicious selection and use of anesthetics. Mitochondrial dysfunction impairs its ability to support oxidative phosphorylation and adenosine triphosphate (ATP) synthesis [1]. Organs with high metabolic demands are principally reliant on mitochondrial function and can exhibit symptoms of dysfunction under stressful conditions [1]. Consequently, the central nervous, cardiovascular, metabolic, and neuromuscular systems are affected [2]. We report a successful anesthetic management of a child with oxidative phosphorylation deficiency type 3 who underwent full mouth rehabilitation.

## Case presentation

A four-year-old child, weighing 11.8 kg, with a history of delayed developmental milestones since early infancy, bilateral sensorineural deafness, hypotonia, and frailness of limbs, was scheduled for full mouth rehabilitation under general anesthesia (GA). At three months of age, the evaluation revealed elevated serum levels of alanine, glutamine, lactate, and ketones. The genetic and metabolic tests confirmed combined oxidative phosphorylation type 3. The child had no history of seizures, lethargy, vomiting, or recurrent respiratory tract infections. There was also a history of sibling death at 75 days of age following fever and seizures. The child underwent bilateral cochlear implantation at one and two years of age under GA, which was uneventful. Additionally, the child had a history of recent (eight months prior) cardiac failure with an ejection fraction (EF) of 28% following respiratory infection, which warranted intensive care unit (ICU) admission and medical management.

During the current admission, routine preoperative blood investigations were normal (Table 1).

**Table 1:** Preoperative investigations.

Parameters	Values	Reference values
Hemoglobin (g/dL)	13.9	13.0-17.0
Total count (cells/cu.mm)	7860	4,000-11,000
Platelet count (lakhs/mm^3^)	3.74	1.5-4.5
Prothrombin time (seconds)	13	10.8-12.9
Control	11.8	
International normalized ratio	1.16	
Partial thromboplastin time (second)	29	22.7-27.5
Control	24.8	
Blood urea nitrogen (mg/dL)	18	7.9-20.1
Creatinine (mg/dL)	0.2	0.5-1.2
Sodium (mmol/L)	139	136-146
Potassium (mmol/L)	4.4	3.5-5.1
Chloride (mmol/L)	110	101-109
Bicarbonate (mmol/L)	17	21-31

An echocardiogram showed left ventricular hypertrophy, grade III diastolic dysfunction, good biventricular function, and an EF of 76%. The patient was on T. Carvedilol 3.125 mg, T. Pyridoxine 50 mg, T. Co-enzyme Q 25 mg, and Syp Levocarnitine 2.5 mg, all of which were continued on the day of surgery.

On examination, the child was alert, with a heart rate of 107 beats per minute, blood pressure of 90/60 mmHg, oxygen saturation (SpO2) of 99%, and a respiratory rate of 18 breaths per minute. The motor power was 4/5 in upper limbs and 3/5 in lower limbs, with good head control and no signs of cardiac failure. The patient was accepted under the American Society of Anesthesiologists - Physical Status (ASA - PS) III under GA. The established fasting guidelines for solid foods were followed precisely, and the child was allowed to take small sips of water up until one hour before the surgical procedure.

Pre-induction monitors included ECG, non-invasive blood pressure (NIBP), SpO2, and temperature. The intravenous line was secured following induction of anesthesia with sevoflurane in oxygen at 6 L/minute. The airway was secured via the nasal route using a flexometallic tube size 4 after intravenous Fentanyl 10 mcgs, Propofol 5 mg, and Atracurium 3 mg. The maintenance of anesthesia was done with air, oxygen, and Sevoflurane at a minimum alveolar concentration (MAC) of 1 and FiO2 (fraction of inspired oxygen) of 0.4. Dextrose 5% at 30 mL/hour was infused with periodic monitoring of blood sugar values. Saline-soaked throat pack was kept in situ before the start of surgery. In addition to standard ASA monitoring, intra-arterial blood pressure monitoring was instituted given the history of cardiac failure and for monitoring metabolic parameters with arterial blood gas analysis (Table 2). Multimodal analgesia was provided with intravenous Fentanyl and Paracetamol. The surgical procedure lasted for 2 hours 40 minutes with minimal blood loss. 

**Table 2:** Intraoperative arterial blood gas (ABG) values.

Parameters	Values	Reference values
pH	7.40	7.35-7.45
PaCO_2 _(mmHg)	31.5	32.0-48.0
PaO_2 _(mmHg)	243	83.0-108.0
Na^+ ^(mmol/L)	142.1	136.0-145.0
K^+ ^(mmol/L)	3.37	3.50-5.10
Ca^2+^ (mmol/L)	1.093	1.150-1.330
Cl^- ^(mmol/L)	107.1	98.0-107.0
Lactate (mmol/L)	1.52	0.20-1.80
Glucose (mg/dL)	98	73.9-100.9
HCO_3_^-^	22	-

At the end of the procedure, neuromuscular blockade was reversed with intravenous neostigmine 0.6 mg and glycopyrrolate 0.2 mg, and after adequate ventilatory efforts and wakefulness, the child was extubated. Oral intake was restarted two hours post-surgery, and the child was subsequently discharged from the Post-Anesthesia Care Unit (PACU).

## Discussion

Mitochondrial disorders have an incidence of 1 in 4 to 5,000 live births and present as multisystem disorders, affecting the central nervous, cardiovascular, muscular, and gastrointestinal systems with varying magnitudes of dysfunction [1,3]. Patients with this condition often require anesthesia for diagnostic purposes and surgical correction of skeletal and neurological deformities. These patients are unable to adapt to stressful conditions demanding high energy requirements resulting in respiratory failure, cardiac depression, conduction abnormalities, seizures, and metabolic encephalopathy [4,5]. Although our child had uneventful general anesthesia for cochlear implant surgery twice, there was a history of cardiac failure secondary to respiratory infection in the recent past, highlighting the unpredictable response to stressful situations.

Our main goal during the perioperative period was to minimize energy demands on the mitochondrial chain, which was achieved by administrating titrated doses of anesthetic agents. Additionally, to mitigate risks associated with prolonged fasting a liberal intake of fluids was permitted until one hour before induction of anesthesia. Excessive crying due to hunger, hypoglycemia, hypovolemia-related sympathetic stimulation, and anxiety leads to high energy requirements paving the way for alternate substrates (fatty acids) for ATP production. However, in patients on ketogenic diets for seizure management, it is imperative to avoid glucose-containing intravenous fluids. Such patients often have difficulty with processing large quantities of glucose and may develop hyperglycemia, if glucose administration exceeds normal maintenance levels [1,6]. Since our child was not on a ketogenic diet, we administered a dextrose-containing solution for intraoperative maintenance therapy with frequent blood glucose monitoring.

Metabolic encephalopathy develops secondary to hypothermia, shivering, hyperthermia, and hypoglycemia. So, we ensured normothermia by monitoring temperature throughout surgery along with convective warmers. All volatile anesthetics suppress oxidative phosphorylation, particularly at complex I and coenzyme Q, animal studies have shown mitochondrial toxicity of volatile anesthetics follows the order: halothane > isoflurane > sevoflurane [7]. In contrast to intravenous anesthetics, which rely on energy-dependent metabolic processes for elimination, volatile anesthetics are predominantly exhaled, which is advantageous. So, we opted for sevoflurane for both induction and maintenance of anesthesia which also has the least metabolism in vivo compared to other agents with cardio stability [1,8].

Propofol inhibits mitochondrial function at multiple levels by affecting complex I, and complex IV, the transport of long-chain acylcarnitine esters and fatty acid transport [1,9]. This can make patients with mitochondrial defects particularly susceptible to developing lactic acidosis, rhabdomyolysis, and lipidemia, leading to cardiovascular collapse and death [1,10]. Continuous propofol infusion specifically inhibits the transport of long-chain acylcarnitine esters, resulting in these life-threatening complications rather than a single bolus dose. Hence, we used a lower dose of propofol along with sevoflurane before intubation. We selected atracurium to avoid the risk of residual neuromuscular blockade that could exacerbate respiratory acidosis in a child with preexisting motor weakness and hypotonia, because of its organ-independent metabolism.

Narcotics have minimal impact on mitochondrial function, but morphine should be used with caution given its profound respiratory depressant effects in patients with preexisting myopathy and compromised respiratory function [1,11]. The effect of anesthetics in mitochondrial disorders is not entirely due to the direct toxic effects on the respiratory chain. It is a summative effect related to the intensity of multisystem dysfunction, duration, and type of surgery evoking stress response. The uneventful perioperative course in our child can be attributed to meticulous planning, judicious of anesthetic agents, and minor surgical procedures all of which avoided metabolic decompensation.

## Conclusions

From an anesthesiologist’s perspective, the primary focus is to maintain the metabolic physiology without major stress on the mitochondrial chain. This is achieved by careful perioperative planning and the use of anesthetics tailored to the individual patient and surgery-centric priorities. Vigilant monitoring of both physiological and metabolic parameters coupled with appropriate choice and dosing of anesthetic agents will aid in safe perioperative management.
